# THE ROLE OF MEDICAID HOME- AND COMMUNITY-BASED SERVICES IN USE OF MEDICARE POST–ACUTE CARE

**DOI:** 10.1093/geroni/igad104.1500

**Published:** 2023-12-21

**Authors:** R Tamara Konetzka, Sijiu Wang

**Affiliations:** University of Chicago, Chicago, Illinois, United States; University of Chicago, Chicago, Illinois, United States

## Abstract

Medicaid-funded long-term services and supports are increasingly provided through home and community-based services (HCBS) to promote continued community living. While an emerging body of evidence examines the direct benefits and costs of HCBS, there may also be unexplored synergies with Medicare-funded post-acute care (PAC). This study aims to provide empirical evidence on how the use of Medicaid HCBS influences Medicare PAC utilization among the dually enrolled. Using national Medicare claims, Medicaid claims (TAF), MDS, and OASIS data from 2014 to 2017, we estimated the relationship between prior Medicaid HCBS use and PAC utilization in a national sample of duals with qualifying index hospitalizations. We used inverse probability weights to create balanced samples on observed characteristics and estimated multivariable regression with hospital fixed effects and extensive controls. We also conducted stratified analyses for key subgroups and tested for interactions between HCBS use and three measures of states’ HCBS generosity. We found HCBS use was associated with a 5.2 percentage-point increase in use of any Medicare-funded PAC, a 7.2 percentage-point increase in home health use, a 2.1 percentage-point decrease in institutional PAC, and a 1.95-day reduction of skilled nursing facility length of stay. In other words, use of Medicaid-funded HCBS was associated with a shift in Medicare-funded PAC use toward home-based settings. These findings were stronger in states with more generous HCBS funding. As we found that use of Medicaid-funded HCBS may alter choices about Medicare-funded PAC use, this synergy with HCBS should be considered by policymakers when contemplating further HCBS expansions.

